# A complex pattern with hexagonal lattice and white-eye stripe in dielectric barrier discharge

**DOI:** 10.1038/s41598-018-21855-3

**Published:** 2018-03-01

**Authors:** Lingyan Wei, Lifang Dong, Weili Fan, Fucheng Liu, Jianyu Feng, Yuyang Pan

**Affiliations:** College of Physics Science and Technology, Hebei University, Baoding, 071002 People’s Republic of China

## Abstract

A novel type of white-eye pattern in a dielectric barrier discharge system has been investigated in this paper. It is a superposition of a hexagonal lattice and a white-eye stripe in appearance and evolves from a white-eye square grid state with the applied voltage increasing. Its spatio-temporal dynamics obtained by an intensified charge-coupled device shows that it consists of three transient rectangular sublattices. The spatiotemporally resolved evolutions of the molecular vibrational temperature and electron density of the pattern are measured by optical emission spectra. The evolution of surface charge distribution is given and its effect on the self-organized pattern formation is discussed.

## Introduction

Patterns, a kind of self-organizing phenomenon, occur in a wide range of natural contexts. Pattern formation has been studied in many different experimental systems^1–27^, such as Faraday system^7–10^, reaction-diffusion system^11–13^, nonlinear optical system^14–16^ and the dielectric barrier discharge (DBD) system^18–27^. Despite the physical differences between these systems, the patterns that appear have common features. In recent years, the study of pattern formation in the DBD system has attracted widespread attention for its many advantages. First of all, a wide variety of patterns have been found in DBD because of its high nonlinear degree. Secondly, the spatio-temporal dynamics of patterns can be easily measured by experimental method due to its self-luminous characteristics. Moreover, it has wide application prospects, such as information processing, the local growth of materials, and plasma photonic crystals^28,29^.

The patterns can be categorized as the simple pattern and the complex pattern. Eyed patterns are a kind of complex pattern which include a special spatial structure like the naked eyes. As a significant kind of pattern types, the eyed pattern has been studied in many systems. For example, G. H. Gunaratne *et al*. gave evidence for the ‘black eye’, a simple hexagonal superlattice Turing pattern, in the chlorine dioxide-iodine-malonic acid (CDIMA) reaction^30^. In nonlinear optical system, M. A. Vorontsov *et al*. observed the black-eye pattern which originates from interactions between spatial modes belonging to different instability bands^31^. L. F. Dong’s group has observed several white-eye patterns in a DBD system, such as the white-eye hexagonal superlattice pattern^32^ and the white-eye square superlattice pattern^33^. Generally, the shapes of sublattices are mainly the same as the global pattern in the previous studies. For example, the ‘black eye’ pattern is a hexagonal superlattice pattern which consists of two hexagonal sublattices (black spots and white circles). The above white-eye superlattice patterns studied in DBD system are composed of three hexagonal or four square sublattices respectively. Here, a complex pattern with hexagonal lattice and white-eye stripe (CPHW) has been reported, whose sublattices’ shapes are not same as the global pattern. It is an interleaving of three rectangular transient sublattices, which is observed for the first time.

Up until now, the formation mechanism of pattern in DBD has not been well known because of its complexity. As is well known, the discharges in gas gap result in the deposition of wall charges on the surface of the dielectrics which will initiate the next discharge. Therefore, the self-organization of the pattern is indeed a self-consistent process of the discharges and the wall charges. In order to get to know the formation mechanism of pattern, the discharge and the wall charges should be measured simultaneously. Recently, the plasma states of the pattern in DBD have been studied by spectroscopic methods^34–36^. J. H. Choi *et al*. found that the decrease in temperature induces a transition of the discharge mode by measuring the global emission spectra of He cryo-DBD^35^. L. F. Dong *et al*. studied the electron density of an individual microdischarge channel in different stable patterns (a square and a hexagon pattern) using a spectral line profile method^36^. In this work, the spatiotemporally resolved plasma parameters (including the molecular vibrational temperature and electron density) are measured by optical emission spectra and the spatio-temporal distribution of the wall charges are given based on the estimation of the electron density for the first time.

In this paper, a CPHW in a DBD system has been observed for the first time. The spatio-temporal dynamics of CPHW and white-eye square grid state (WESGS) are given. The spatiotemporally resolved evolutions of the molecular vibrational temperature and electron density of CPHW are measured by optical emission spectra. The spatio-temporal distribution of the wall charges is given and its effect on the self-organization of the pattern is discussed.

## Results

### The evolution of the discharge pattern

**Figure 1:**
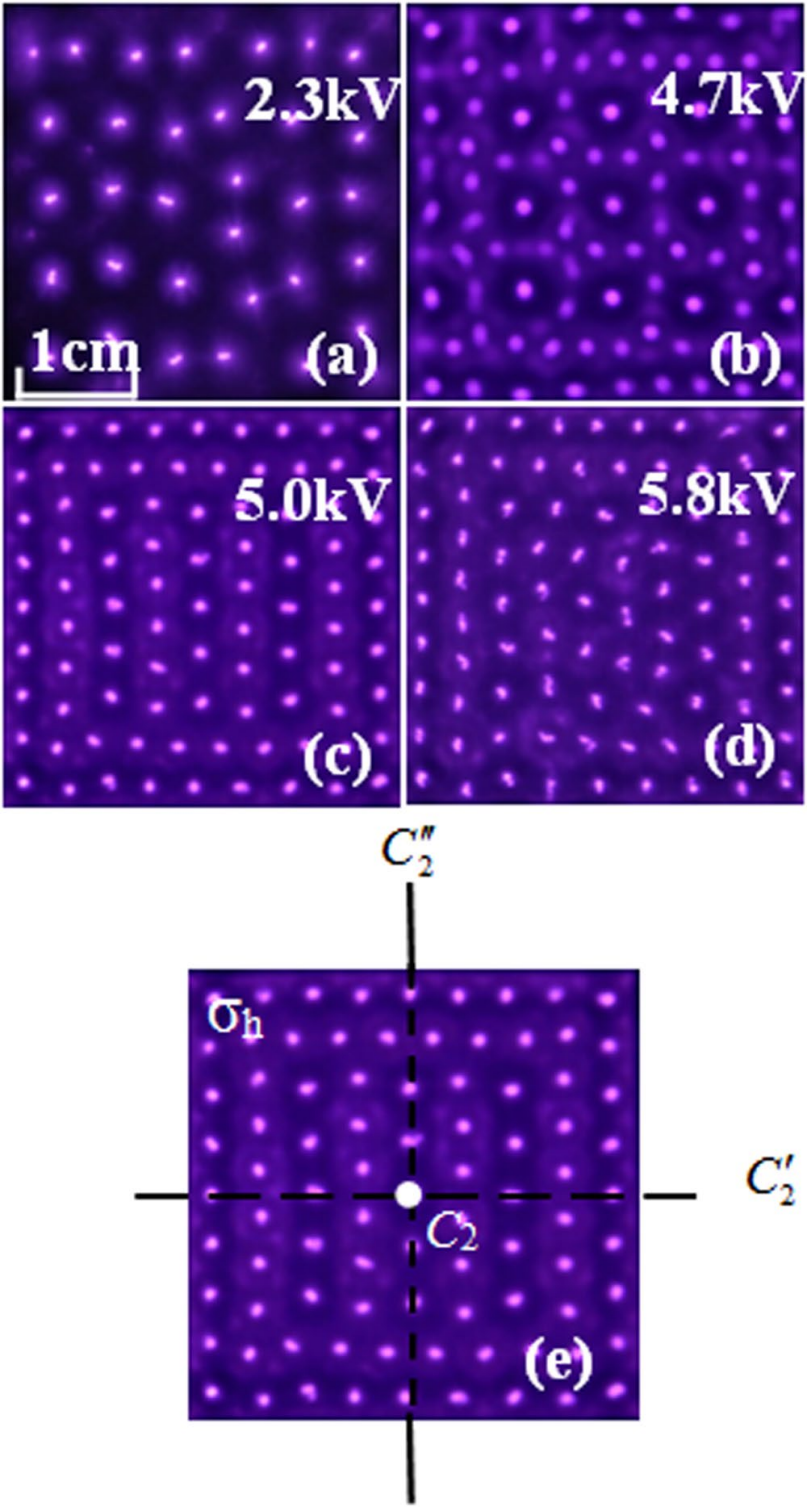
Evolution of patterns with increasing voltage. (**a**) Random filaments, *U* = 2.3 kV. (**b**) White-eye square grid state, *U* = 4.7 kV. (**c**) Complex pattern with hexagonal lattice and white-eye stripe, *U* = 5.0 kV. (**d**) Chaotic state, *U* = 5.8 kV. (**e**) The symmetric operations of (**c**). Other experimental parameters: gas pressure *p* = 40 kPa, argon concentration *φ* = 50%, driven frequency *f* = 60 kHz, gas gap *d* = 3.0 mm, exposure time of the pictures *t* = 40 ms.

Figure 1(a–d) show the images of the pattern scenario with the applied voltage increasing. As shown in Fig. 1(c), the CPHW appears at 5.0 kV. With the increasing of the applied voltage, the pattern bifurcates from random filaments (2.3 kV), white-eye square grid state (WESGS) (4.7 kV) and finally to a chaotic state (5.8 kV). It can be seen that the CPHW is the compound of hexagonal lattice and white-eye stripe in appearance. The CPHW which has not been found in any other system enriches the pattern species.

In order to characterize the symmetry of the pattern, the corresponding point group and symmetric operations of patterns in Fig. 1(b) and (c) are analyzed. It can be seen that the WESGS belong to dihedral group *D*_4*h*_, and the symmetric operations are *E*, 5*C*_2_, *C*_4_, ${C}_{4}^{3}$, *σ*_*h*_, *S*_4_, ${S}_{4}^{3}$, ἰ, and 4*σ*_*v*_, respectively. The CPHW belong to dihedral group *D*_2*h*_, and symmetric operations are *E*, 3 *C*_2_, *σ*_*h*_, ἰ, and 2 *σ*_*v*_, respectively (two cyclic symmetries 2*C*_2_ in the principal plane, one *C*_2_ perpendicular to the principal plane, and one horizontal mirror plane *σ*_*h*_ as shown in Fig. 1(e). And one identity symmetry element *E*, one inversion symmetry element ἰ, and two vertical mirror planes 2 *σ*_*v*_).

### Discharge characteristic measurement of CPHW and WESGS

**Figure 2:**
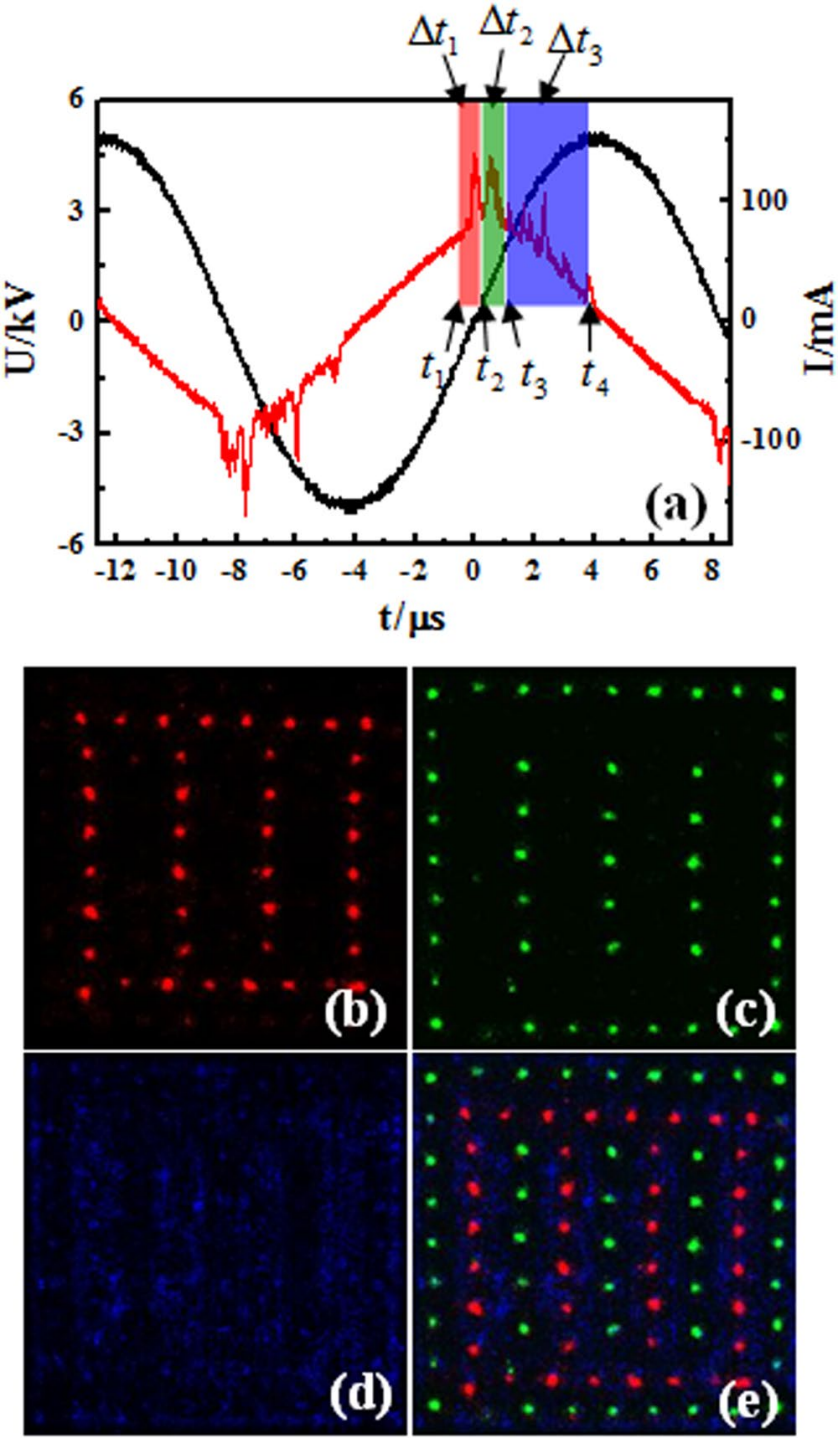
Instantaneous images of the complex pattern with hexagonal lattice and white-eye stripe. (**a**) Waveforms of the applied voltage and current of the pattern. (**b**) Big spots. (**c**) Small spots. (**d**) Halos. The pictures (**b**–**d**) are, respectively, correlated with current pulse phases (Δ*t*_1_ = 500 ns, = 400 ns, and = 1900 ns) in (**a**) and integrated over 100 voltage cycles to obtain sufficient light signals. (**e**) The frame is the superposition of (**b**,**c** and **d**).

To explore the formation mechanism of CPHW, the instantaneous images with different exposure time are taken by an ICCD camera. Figure 2(a) presents the waveforms of the voltage and the current. Correlated with the current pulse phases (Δ*t*_1_, Δ*t*_2_, and Δ*t*_3_) denoted in Fig. 2(a), instantaneous images are recorded and shown in Fig. 2(b–d), and the pictures which are integrated over 100 voltage cycles make the structures of the pattern clear. The four pictures indicate that during the first current pulse (Δ*t*_1_), which starts at the negative half voltage cycle and ends at the positive half voltage cycle, the discharge ignites in the big spots’ position. Then another rectangular sublattice (small spots) is generated in Δ*t*_2_. Finally the halos discharge in the third part of the current (Δ*t*_3_). Figure 2(e) is the superposition of (b), (c) and (d). The results show that the CPHW is composed of three transient rectangular sublattices: big spots, small spots and halos (denoted by B, S and H, respectively), and the discharge sequence is B-S-H-B-S-H within one cycle of the voltage. These three transient rectangular sublattices all belong to *D*_2*h*_ point group. The global structure also belongs to *D*_2*h*_ point group although the global structure of CPHW is the compound of a hexagonal lattice and a white-eye stripe. It can be seen that the sublattices show the intrinsic symmetry feature of the global pattern.

**Figure 3:**
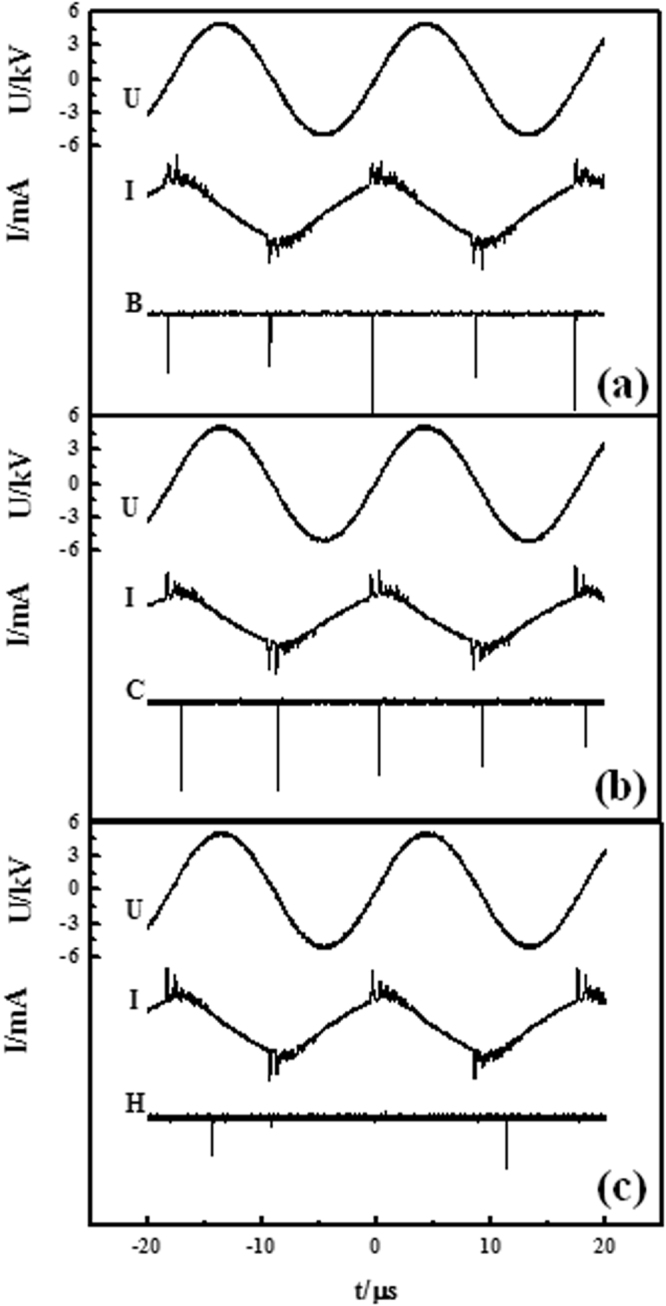
Time correlation measurement of the white-eye square grid state. B stands for the big spots in the center of the halos, C stands for the central spots, H stands for the halos. (**a**) Time correlation measurement of big spots. (**b**) Time correlation measurement of central spots. (**c**) Time correlation measurement of halos. *U* stands for voltage curve, *I* stands for current curve.

In order to study the symmetry changes in the pattern evolution process, the temporal correlations between the filaments of WESGS are measured by a PMT. Figure 3 gives the light emissions of big spot, central spot, and part of halo (denoted by B, C, and H) respectively. It is found that the big spots with halos around are ignited simultaneously corresponding to the first pulse phase (the falling edge of the voltage) in each half driving cycle in Fig. 3(a). The central spots discharge slightly later (the rising edge of the voltage) than the big spots (Fig. 3(b)), and the halos discharge at the last part (the rising edge of the voltage) of the pulse phase (Fig. 3(c)). When a part of the halo is measured by PMT, the optical signal only appears in some half voltage cycle (Fig. 3(c)), indicating that the halo consist of several filaments (generally 3–4 filaments^37^) whose positions are not fixed. By analogy, the halo of CPHW also consists of unfixed filaments. Thus, it can be obtained that the WESGS is an interleaving of three square sublattices, and the discharge sequence is big spots–central spots–halos in each half voltage cycle. The WESGS and its sublattices all belong to *D*_4*h*_ point group.

**Figure 4:**
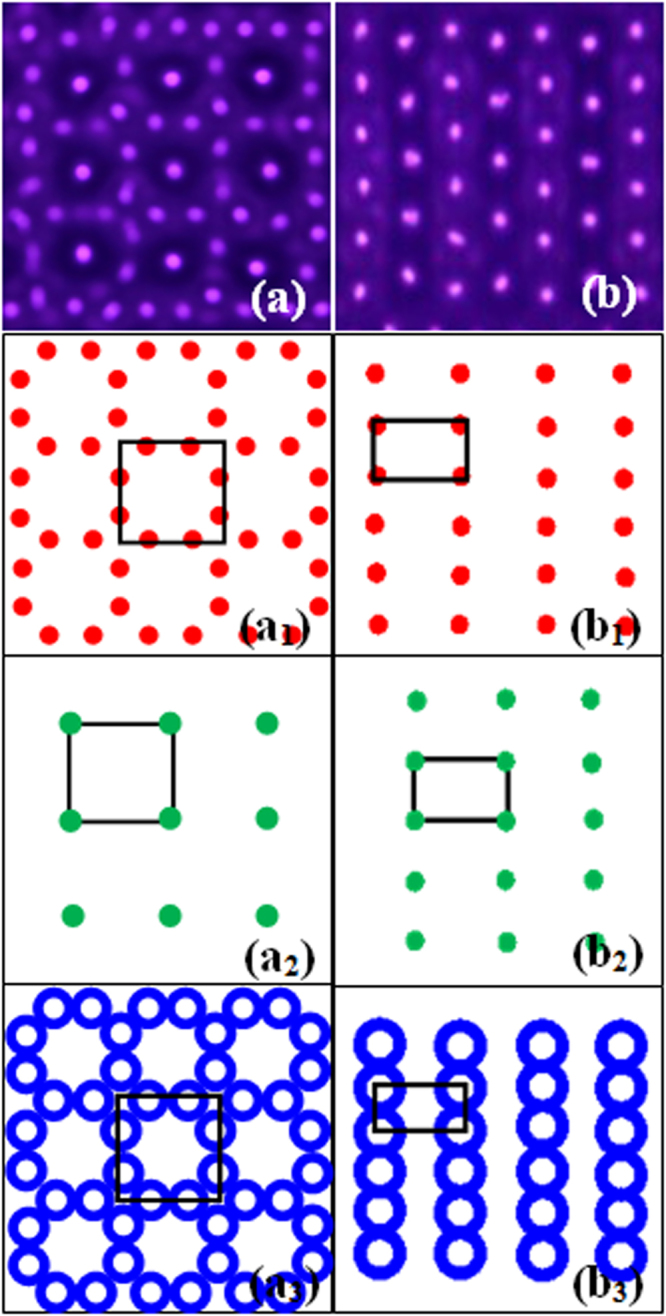
Schematic diagrams of the white-eye square grid state and the complex pattern with hexagonal lattice and white-eye stripe. (**a**) The white-eye square grid state. (a_1_–a_3_) Are the different sublattices of the white-eye square grid state. (**b**) The complex pattern with hexagonal lattice and white-eye stripe. (b_1_–b_3_) Are the different sublattices of the complex pattern with hexagonal lattice and white-eye stripe.

The comparison between the CPHW and the WESGS is shown in Fig. 4. It visually shows that the WESGS is an interleaving of three different square sublattices, and all of the sublattices (Fig. 4(a1–a3)) show the square symmetry. And the global CPHW is the compound of hexagonal lattice and white-eye stripe, but the three sublattices of CPHW all present a rectangular shape (Fig. 4(b1–b3)). In a word, both CPHW and WESGS have three sublattices, but their symmetry decreases from *D*_4*h*_ to *D*_2*h*_ through self-organization.

### The spatiotemporally resolved OES of the CPHW

**Figure 5:**
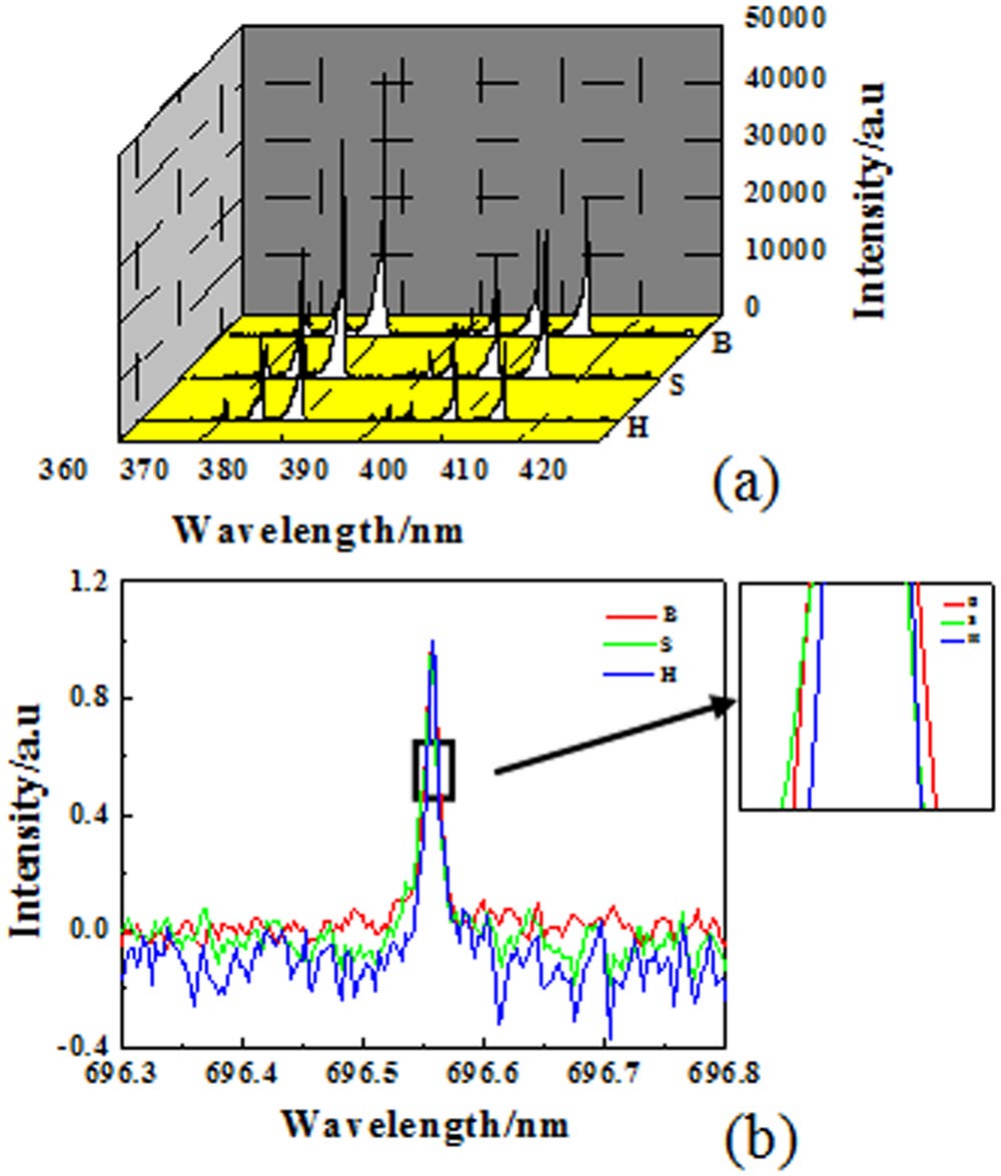
(**a**) Spectrum lines of the broadenings of N_2_ second positive band system (*C*^3^*П*_*u*_ → *B*^3^*П*_*g*_) at different positions. (**b**) The profiles of the spectral line 696.5 nm of the three discharges sublattices.

In order to study the influence of the plasma states on the pattern’s self-organization process, the plasma parameters are studied by optical emission spectra (OES) (Fig. 5). As each sublattice of the CPHW discharges in the corresponding current pulse, the OES collected from any sublattice should also represent the OES of the pattern discharge during the corresponding current pulse. Therefore, the spatially resolved OES measurements of the three sublattices can also achieve the temporally resolved measurements, i.e., the spatiotemporally resolved OES of the CPHW can be obtained by collecting OES from three sublattices respectively.

In experiments, the OES of each sublattice is collected by using a spectrometer with an optical fiber probe. For measuring the spectrum of an individual microdischarge channel in each sublattice, the discharge pattern is magnified by a lens so that the diameter of the image of one microdischarge channel is little more than inside diameter of optical fiber probe, making sure that the emission of only one microdischarge channel is transmitted into the optical fiber probe.

Figure 5(a) gives the spectrum lines of the N_2_ second positive band system (*C*^3^*П*_*u*_ → *B*^3^*П*_*g*_) of three sublattices, from which the molecular vibrational temperatures of three sublattices are calculated. After a lot of measurements, the average vibrational temperatures are (2390 ± 30) K for B, (2440 ± 30) K for S, and (2650 ± 30) K for H, respectively. The ascending order of the temperature is B-S-H.

Figure 5(b) shows the profiles of Ar I (2*P*_*2*_ → *1S*_5_) spectral line of the three different sublattices, which are obtained by selecting the 2400 groove/mm grating. Results indicate that the broadening of B, S and H is (0.014 ± 0.004) nm, (0.013 ± 0.004) nm, (0.011 ± 0.004) nm, respectively. As is well known, in plasma with electron density higher than 10^15^ cm^−3^, the Stark effect plays an important role in the atom spectral line broadening. The electron density *N*_*e*_ can be estimated by Stark broadening of Ar I line *ω*_*t*_ through

$${\omega }_{t}=2\times [1+1.75\times {10}^{-4}\,{{N}_{e}}^{1/4}\alpha \times (1-0.068\,{{N}_{e}}^{1/6}\,{{T}_{e}}^{-1/2})]\times {10}^{-16}{\omega }_{e}{N}_{e}$$

where *T*_*e*_ (in kelvins) is the electron temperature, *ω*_*e*_ is the electron impact width, and *α* is the ion broadening parameter^38^. So it can be approximately obtained that the ascending order of the electron density is H-S-B.

**Figure 6:**
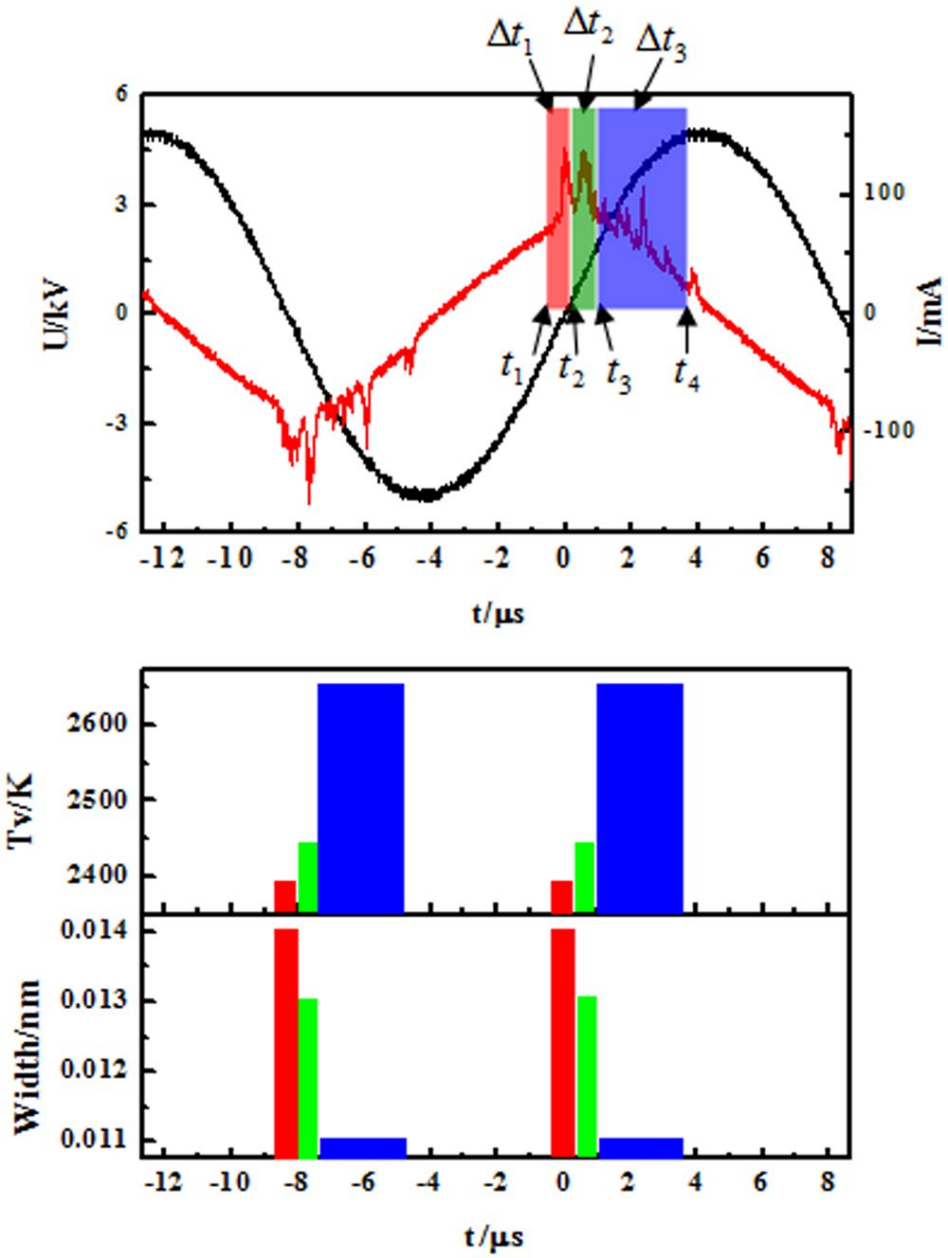
The spatiotemporally resolved plasma parameters of the complex pattern with hexagonal lattice and white-eye stripe.

Figure 6 shows the spatiotemporally resolved plasma parameters of the complex pattern with hexagonal lattice and white-eye stripe. It can be summarized that the big spots (B) corresponding the first pulse (Δ*t*_1_) have the lowest molecular vibrational temperature and the largest electron density. The small spots (S) corresponding the second pulse (Δ*t*_2_) have moderate molecular vibrational temperature and electron density, while the halos (H) corresponding the third pulse (Δ*t*_3_) have the highest molecular vibrational temperature and the smallest electron density. In this way, it obtains the spatiotemporally resolved plasma parameters of CPHW. The results show that the three sublattices of CPHW are in different plasma states. Generally, the surface density of wall charges can be characterized by the electron density since the discharge duration of a filament in different sublattices is almost the same. So it can be obtained that the ascending order of wall charges is H-S-B.

By comparing Fig. 2(c) and (d), it can be seen that different plasma states can lead to different self-organization processes. As mentioned above, the small spots and halos are in different plasma states. The filaments in Fig. 2(c) are fixed, while the filaments in halo in Fig. 2(d) are random. In other words, filaments of halo can not form a regular sublattice in each half cycle, but they can form regular halo sublattice in hundreds cycles. Thus the small spots sublattice is the fixed arrangement by self-organization, while the halos sublattice is the statistically outcome of self-organization. In a word, different plasma states can lead to different self-organization processes.

## Discussions

**Figure 7:**
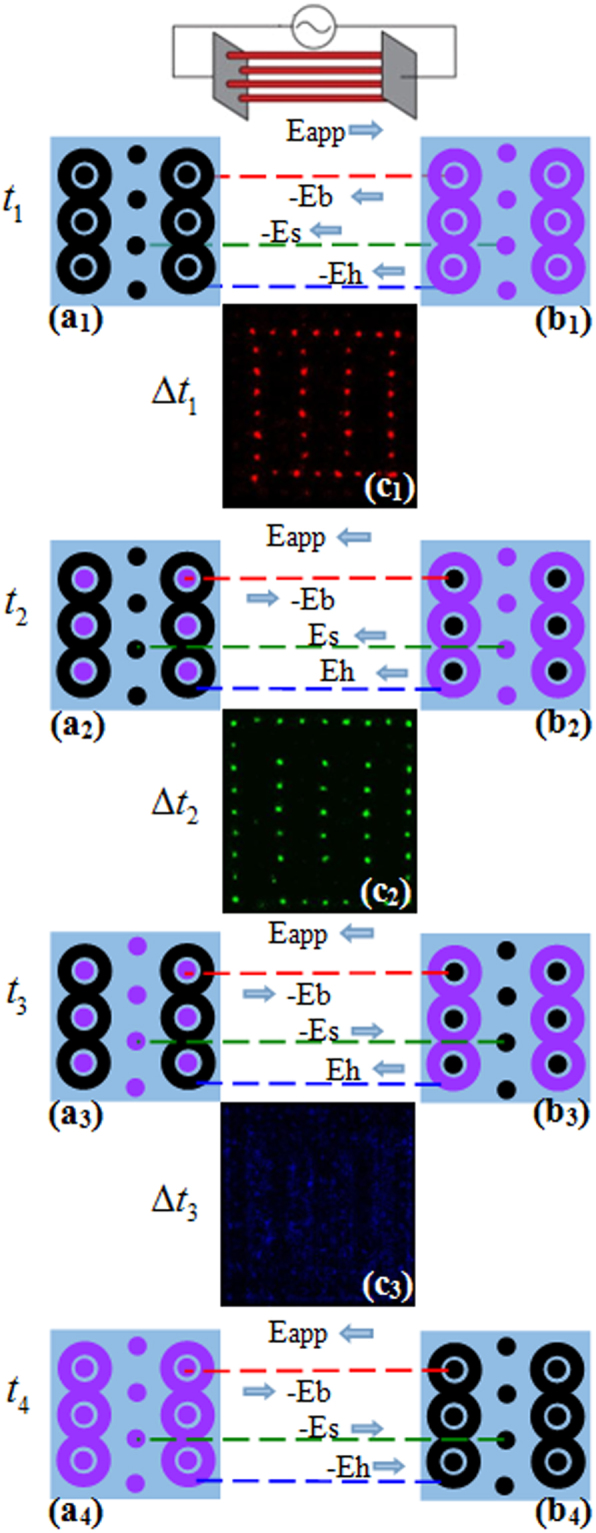
Time-resolved evolution sequence of the complex pattern with hexagonal lattice and white-eye stripe. (a_1_–a_4_) One cell of the wall charges’ sublattice at different times on one side of dielectric surface. (b_1_–b_4_) One cell of the wall charges’ sublattice at different times on the other side of dielectric surface (red represents the positive charge, black represents the negative charge). (c_1_–c_3_) Images corresponding over a period of time are denoted in Fig. 2(a). (c_1_–c_3_) Are pictures taken by ICCD camera which are integrated over 100 voltage cycles. A simplified circuit is given in the upper part of this figure. The applied electric field and the internal electric fields induced by big spots, small spots and halos are named as *E*_app_, *E*_b_, *E*_s_ and *E*_h_, respectively. The negative sign is added to *E*_b_, *E*_s_ and *E*_h_ when they are reversed to *E*_app_.

According to the above results, the spatio-temporal characteristics of the CPHW are analyzed in Fig. 7. The wall-charge electric fields of the big spots, small spots and halos are named *E*_*b*_, *E*_*s*_ and *E*_*h*_, respectively. The negative sign is added to *E*_*b*_, *E*_*s*_ and *E*_*h*_ when they are reversed to *E*_*app*_. The different discharge moments (*t*_1_ − *t*_4_) are marked in Fig. 7(a). At the end of one half voltage cycle (*t*_1_), the cell of the wall charges is shown in Fig. 7(a1) and (b1) (red represents the positive charge, black represents the negative charge). It indicates that the wall-charge electric fields of all spots (−*E*_*b*_, −*E*_*s*_, and −*E*_*h*_) have an opposite direction with *E*_*app*_, and the sum fields is large enough to overcome applied voltage to reach the breakdown threshold of the big spots who have the largest wall charges. Then the big spots are reversed ignition (Fig. 7(c1)). After the big spots discharge, the status of wall-charge electric fields turns into Fig. 7(a2) and (b2). It is worth noting that *E*_*app*_ have reversed in *t*_2_, so did the wall-charge electric fields. The discharge continues until the rising edge of voltage which ensures the reversal of big spots’ wall charges. In this discharge process, all of the wall-charge electric fields act as an activator, and the applied electric field acts as an inhibitor in the process of this discharge. Then under the influence of *E*_*app*_, −*E*_*b*_ and *E*_*s*_, the small spots who have more wall charges than halos are ignited firstly at the rising edge of applied voltage with the voltage increasing (Fig. 7(c2)). Due to the feedback electric field is generated by wall charges accumulated on the dielectric surface, the sum of fields (*E*_*app*_ and −*E*_*s*_) tends to decrease, which leads to discharge quenching. Each cell of the wall charges at *t*_3_ is shown in Fig. 7(a3) and (b3). After the small spots discharge, the wall charges accumulated in the place of the small spots have a counteractive effect on surrounding. Under the effects of *E*_*app*_, −*E*_*b*_, and −*E*_*s*_, the halos discharge far away from the small spots and form a shape of ring on the outside of the big spots (Fig. 7(c3)). After the halos discharge, the wall charges will be the same as that in Fig. 7(b4) and (a4). Then the applied voltage begins to decrease, and the pattern will repeat the above sequence which demonstrates the periodicity of the sequence. It’s worth noting that the applied voltage plays a leading role in the discharging of small spots and halos, while the discharging of big spots is mainly effected by wall charges.

Above all, a CPHW whose sublattices’ shapes are different from the global pattern has been obtanied. The CPHW is an interleaving of three rectangular transient sublattices, and the discharge sequence is big spots–small spots–halos in each half voltage cycle. The spatiotemporally resolved plasma parameters of CPHW is obtained, and each discharge sublattice presents different plasma states. The plasma states affect the self-organization of the pattern.

In conclusion, a novel type of white-eye pattern which consists of three rectangular sublattices is first reported in a dielectric barrier discharge system. The complex pattern is the compound of a hexagonal lattice and a white-eye stripe in appearance. The spatio-temporal characteristics of the complex pattern show that the CPHW consists of three different rectangular sublattices. It demonstrates that the shapes of sublattices are different from that of the global structure in the complex pattern. The spatiotemporally resolved evolutions of the molecular vibrational temperature and electron density of the pattern are measured by optical emission spectra. The evolution of wall charges distribution is given and its effect on the self-organization of the pattern is discussed. It is found that the plasma states affect the self-organization of the pattern. These results give insights on the underlying physics of the formation mechanism of the complex patterns in DBD.

## Methods

### Experimental setup

**Figure 8:**
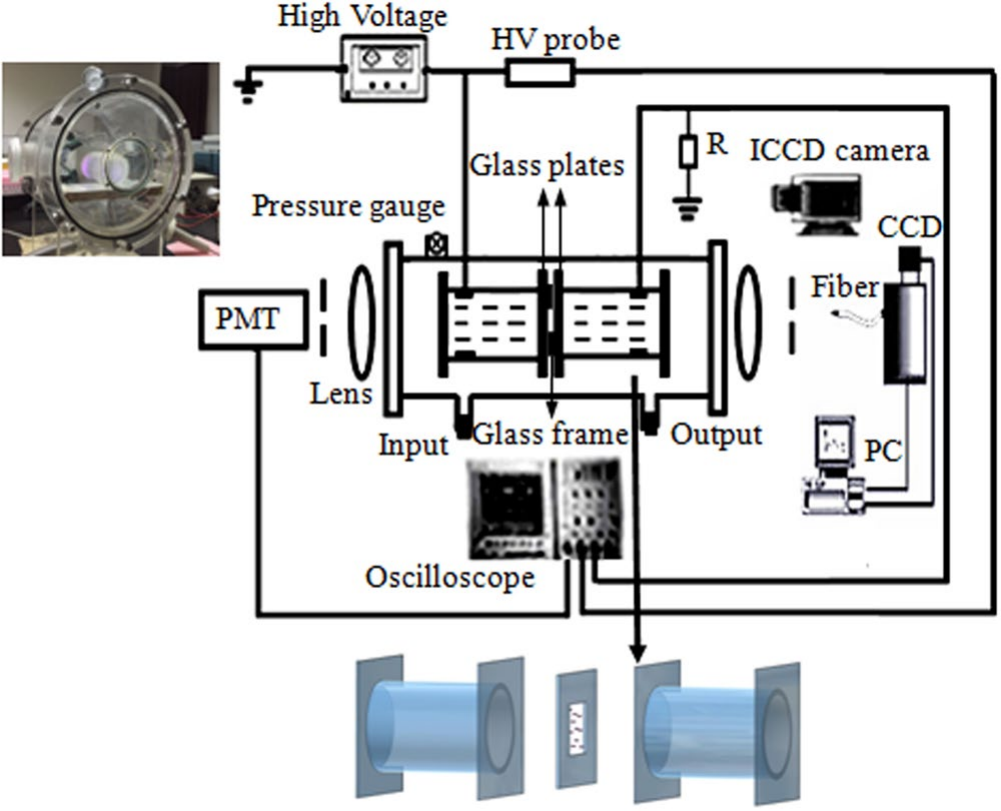
Schematic diagram of the experimental setup.

The schematic diagram of the experimental setup is shown in Fig. 8. The electrodes are two cylindrical containers which are sealed with 1.5 mm thick glass plates and filled with water. A metallic ring which is immersed in each container is used for connecting to a sinusoidal ac power supply. The ac power supply (Nanjing, China, Maoer Electronics Co., Ltd, CTP-2000K) has a fixed frequency of 60 kHz. A square glass frame with a 3 mm thickness is clamped between the two parallel glass plates, serving as a discharge gap. The whole discharge cell is enclosed in a big container where the pressure controlled in 40 kPa and filled with 50% argon and 50% air. The voltage waveform is detected by probes and recorded by a digital phosphor oscilloscope (Tektronix TDS 3054B). In circuit, the water electrodes are in series with a 50 resistor. A high-voltage probe (Tektronix P6015A 1000×) is used to detect the applied voltage. Another probe (Tektronix TPP1000) is used to detect the voltage across the resistor and the current waveform is calculated by the resistor voltage divided by the resistor. The mainly measuring instruments in this paper are intensified charge-coupled device (HSFC-PRO, 3 channels) camera, photomultiplier (PMT, ET 9085) and spectrometer (Acton Advanced SP 2750 A, CCD: 1340 × 400 pixels), respectively.

With the applied voltage increasing, filaments will form in the square glass frame between the two water electrodes. Different patterns will appear under different applied voltages (other experimental parameters: gas pressure p = 40 kPa, argon concentration = 50%, driven frequency f = 60 kHz, gas gap d = 3.0 mm, exposure time of the pictures t = 40 ms).
